# Integrating an electrically active colloidal quantum dot photodiode with a graphene phototransistor

**DOI:** 10.1038/ncomms11954

**Published:** 2016-06-17

**Authors:** Ivan Nikitskiy, Stijn Goossens, Dominik Kufer, Tania Lasanta, Gabriele Navickaite, Frank H. L. Koppens, Gerasimos Konstantatos

**Affiliations:** 1 ICFO—Institut de Ciencies Fotoniques, The Barcelona Institute of Science and Technology, Avenida Carl Friedrich Gauss 3, Castelldefels, 08860 Barcelona, Spain; 2 ICREA-Institució Catalana de Recerca i Estudis Avançats, Lluis Companys 23, 08010 Barcelona, Spain

## Abstract

The realization of low-cost photodetectors with high sensitivity, high quantum efficiency, high gain and fast photoresponse in the visible and short-wave infrared remains one of the challenges in optoelectronics. Two classes of photodetectors that have been developed are photodiodes and phototransistors, each of them with specific drawbacks. Here we merge both types into a hybrid photodetector device by integrating a colloidal quantum dot photodiode atop a graphene phototransistor. Our hybrid detector overcomes the limitations of a phototransistor in terms of speed, quantum efficiency and linear dynamic range. We report quantum efficiencies in excess of 70%, gain of 10^5^ and linear dynamic range of 110 dB and 3 dB bandwidth of 1.5 kHz. This constitutes a demonstration of an optoelectronically active device integrated directly atop graphene and paves the way towards a generation of flexible highly performing hybrid two-dimensional (2D)/0D optoelectronics.

## Abstract (web summary)

The combination of fast photo-response and high gain plays a pivotal role in photodetector devices. Here the authors combine a colloidal quantum dot photodiode with a graphene phototransistor to overcome the speed, quantum efficiency and linear dynamic range limitations of available phototransistors.

## Graphical abstract

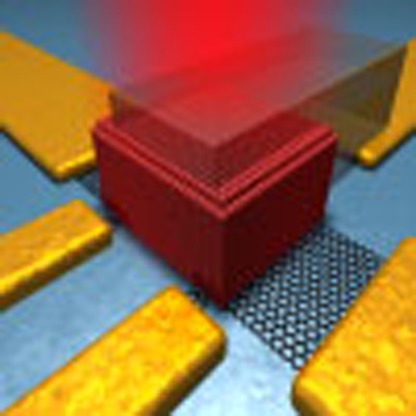

A vast number of applications calls for highly sensitive detectors that can sense light from the ultraviolet to the short-wave infrared (SWIR) range, covering a broad spectrum of 300–3,000 nm^1^. Ideally these detector technologies should be based on CMOS compatible platforms for monolithic integration with read-out electronics to cater for high-density, high-throughput and low-cost manufacturing. Graphene^2^,^3^ and colloidal quantum dots (CQDs)^4^,^5^ are two material platforms that have proved to fulfil those requirements^6^,^7^,^8^,^9^. Essential features for highly sensitive photodetectors is the very high quantum efficiency expressed in the number of primary photo-generated carriers collected per incident photon, the low noise and the presence of an additional amplification mechanism, the gain, which is the number of electrically circulated carriers per incident photon. The electrical output of a photodetector is expressed by its responsivity (in A W^−1^ or V W^−1^) which is proportional to both the quantum efficiency and the gain. These parameters eventually determine the sensitivity of a photodetector quantified via the specific detectivity D*. D* is defined as 
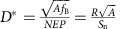
, where *NEP* is the noise equivalent power, *R* is the responsivity, *A* is the optically active area of the detector, *f*_B_ is the noise bandwidth and *S*_n_ is the noise spectral density of the detector.

Graphene's unique properties such as high charge carrier mobility, atomically thin profile and broadband absorption has made this material a very attractive platform for photodetectors^10^. Graphene has rapidly proved to be excellent for ultrafast photodetectors^11^,^12^,^13^. The high carrier mobility enables ultrafast conversion of photons or plasmons to electrical currents or voltages. Yet, graphene's atomically thin profile inhibits efficient optical absorption. Until now, most sensitive graphene-based photodetectors were phototransistors that relied on the presence of an electrically passive sensitizing layer that consisted of CQDs^14^,^15^, perovskites^16^, carbon nanotubes^17^ or other two-dimensional materials^18^,^19^. The passive aspect of the sensitizing layer has thus far limited the quantum efficiency (defined as the number of transferred charges per incident photon), linear dynamic range and speed of the detector. Namely, the external quantum efficiency (*EQE*) of these systems is a trade-off between light absorption, which increases for thicker sensitizing layers, and charge transfer, which decreases for thicker layers as it is limited by carrier diffusion towards the graphene channel. This has limited the quantum efficiency to about 25% for an optimized layer of CQDs of thickness 80 nm^14^, the time response, which is limited by the long trapping times of the sensitizing centres^20^ and the dynamic range, which is determined by the density of the trap-state sensitizing centres^21^.

In this letter, we address these three main challenges by presenting a photodetector architecture that is essentially a graphene–CQD photodiode and a high-gain phototransistor. This is realized by transforming the electrically passive sensitizing layer to an active one, where the electric field in the photodiode significantly enhances charge collection due to carrier drift instead of solely relying on diffusion. Our devices operate in the visible, near-infrared and SWIR range and show an *EQE* as high as 70–80% (limited by reflection), a sub-millisecond temporal response, a gain-bandwidth product on the order of 10^8^ and a linear dynamic range in excess of 110 dB. This highly performing detector also exhibits very high sensitivity with experimentally measured D* of 1 × 10^13^ Jones.

## Results

### Device structure and essence

**Figure 1:**
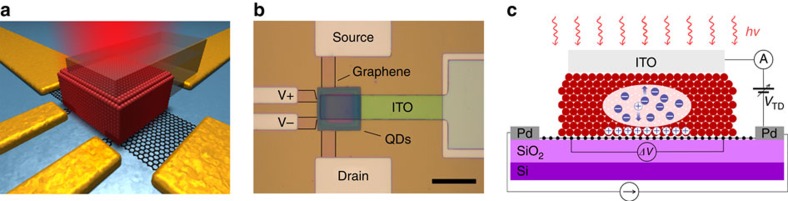
Hybrid graphene–CQDs phototransistor with a transparent top-electrode. (**a**) Schematic of device structure. (**b**) Optical image of a device used in the study. Device elements are labelled. Borders of graphene channel are highlighted in black. Active device area is located between the inner graphene probes, marked as V+ and V−. CQD layer is deposited on the active area and exceeds it in size to ensure its uniform coverage and prevent shorts between the ITO and graphene channel. Scale bar, 30 μm. (**c**) Schematic of phototransistor operation. Incident light creates electron–hole pairs in the CQD layer. Electrons remain in the CQD, while holes, driven by depletion on the interface with graphene, are transferred to the graphene channel and alter its resistance. Constant current is driven between source and drain electrodes, while voltage drop is measured between V+ and V− electrodes. Thus, the photo-induced change in resistance (or voltage drop) is measured with the highest accuracy excluding the resistance of the metal and graphene contacts.

Our photodetector platform integrates a CQD photodiode atop a graphene transistor. The CQD photodiode consists of an indium tin oxide (ITO) top-contact acting as the cathode of the CQD photodiode, whereas graphene acts as the hole acceptor contact and the charge transport channel for the phototransistor. Figure 1a illustrates a schematic of the hybrid graphene transistor—CQD photodiode detector. Figure 1b shows a top-view optical microscopy image of a typical device and the electrodes in use. The device consists of a graphene channel with a patterned 300-nm-thick CQD layer on top, which is overcoated with a top-contact of an ITO electrode. The active area of the photodetector is formed in the overlapping region of the three layers. The CQD layer is patterned to ensure that no charge carriers can escape from it to the drain contacts omitting the graphene. The read-out of the photodetector is then based on the voltage read-out at the sides of this active area, while source and drain electrodes are biased at a constant current (four-terminal sensing). Such device structure has been employed to eliminate the contact resistance from the measurements and to isolate the measurements over the active area of the photodetector from the area not covered by the ITO electrode. The electronic circuit and diagram of the device is illustrated in the side-view schematic of Fig. 1c.

**Figure 2:**
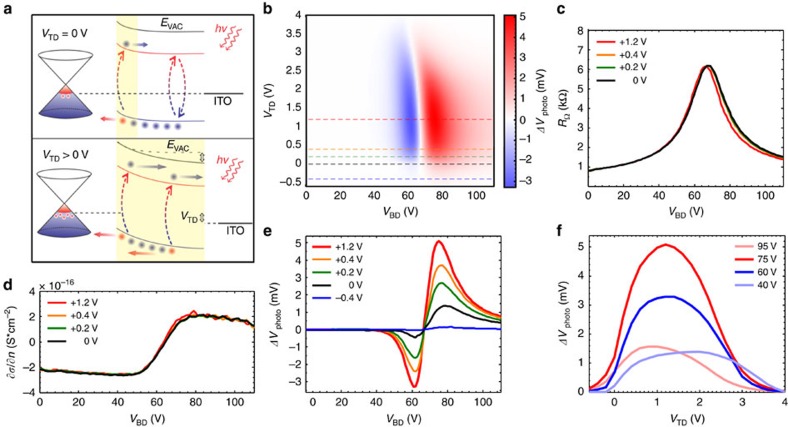
Enhancement of photoresponse using a top-electrode. (**a**) Energy level diagrams of the graphene–CQDs interface without (top) and with (bottom) positive *V*_TD_ voltage applied. Positive *V*_TD_ voltage induces electrical field perpendicular to the graphene–CQDs interface and increases the depletion width and charge collection efficiency. The yellow area qualitatively indicates the change of depletion width on the scale below it. (**b**) Colour map of the photovoltage response as a function *V*_BD_ and *V*_TD_ for 100 mW m^−2^ illumination with a 635-nm light. Positive (red) and negative (blue) area correspond to injection of photo-generated holes into the *n*- and *p*-doped graphene. Dashed lines indicate the positions of cross-sections (**e**). (**c**) Resistance of the device's active area as a function of *V*_BD_ for fixed values of *V*_TD_ specified in the legend. (**d**) Transconductance of the device as of *V*_BD_ for fixed values of *V*_TD_ specified in the legend. Presented curves demonstrate no significant change with increasing *V*_TD_. (**e**,**f**) Photovoltage response, in absolute values, taken from cross-sections of the colour map (**b**) at fixed values of *V*_TD_ (**e**) and *V*_BD_ (**f**) as specified in the legends.

The essence of the device operation is captured in the band structure, shown in Fig. 2a, representing the operation without and with voltage applied on the ITO top-contact relative to drain electrode (*V*_TD_). Considering first operation without *V*_TD_: on carrier photogeneration in the CQD layer, holes are transferred to the graphene via drift from the narrow depletion region formed in the PbS QD solid near the graphene–QD interface and via diffusion further away from the quasi-neutral region. Prior studies have shown that efficient charge collection in this configuration occurs at an optimum thickness of 80–90 nm of PbS QDs, limited by the short carrier diffusion length^22^,^23^. In the case of a passive CQD sensitizing layer, the electrons remain trapped in the CQD solid and significant gain is achieved^14^ as:


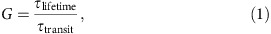


where *τ*_lifetime_ is the carrier lifetime of the trapped electrons in the sensitizing centres of PbS QDs and *τ*_transit_ is the transit time of holes in the graphene channel.

Significant enhancement in charge collection can be foreseen by the application of an electric field by using a transparent top-contact—ITO in this case. The application of *V*_TD_ bias extends the depletion region in the 300-nm-thick CQD layer, amplifying thus the zone of efficient charge collection and concomitantly quantum efficiency. This enhancement is evident in Fig. 2b, which shows a map of the measured photovoltage of the detector as a function of the back-gate bias (*V*_BD_) and the top-contact bias (*V*_TD_), and Fig. 2e,f which represents line traces extracted from the map. The enhancement of the photoresponse on the application of a top-contact bias exceeds a factor of 4 at ∼*V*_TD_=1.2 V, as compared with photoresponse at *V*_TD_=0 V. Further increase of *V*_TD_ results in opposite effects and fully suppresses the photo-induced voltage change at *V*_TD_=4 V. This phenomenon is observed for any *V*_BD_ value for both *n*-doped and *p*-doped graphene channel, and this is due to increased leakage current from the CQD photodiode (as discussed below). It is important to note that changing the potential of the top electrode induces a corresponding change in graphene doping; however, this change is weak and cannot account for the observed change in photocurrent. This is clear in Fig. 2c, which shows the weak dependence of graphene's doping on the applied *V*_TD_ as a result of suppressed gating via the CQD layer. Figure 2d shows the transconductance of the device (∂*g*/∂*n*) extracted from the corresponding resistance curves that is not affected by the electric bias of the top-contact, from which we conclude that the photoresponse enhancement is not due to increasing transconductance (see Supplementary Fig. 1 for more details).

### Photodiode operation

**Figure 3:**
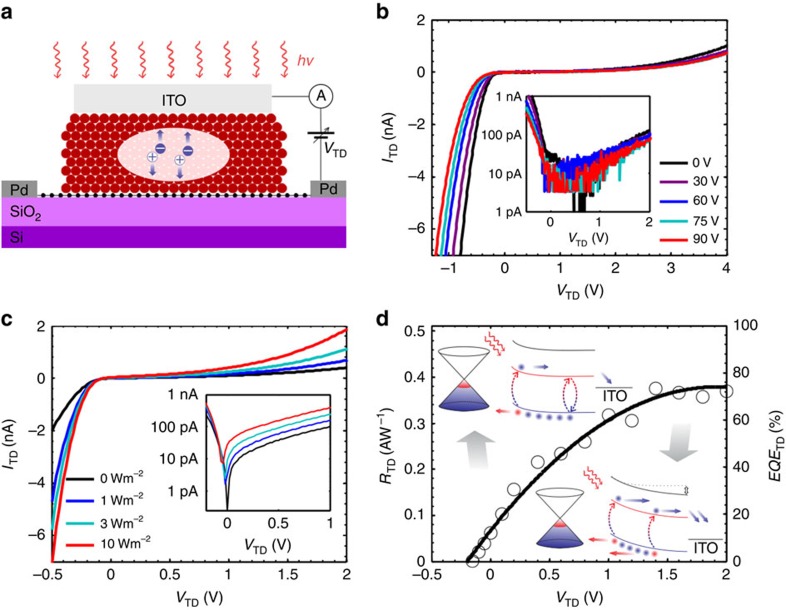
Optoelectronic characterization of the graphene–CQD–ITO photodiode. (**a**) Schematic of graphene–CQDs photodiode operation. Incident light creates electron–hole pairs in the PbS quantum dots, which are drifted by the built-in electric field at the interface and by the bias applied across the diode. Source V+ and V− probes of the device are kept floating while *I*_TD_ current is measured between top and drain probes. (**b**) Current–voltage characteristic of the graphene–CQD diode for gate-voltages *V*_BD_ specified in the legend. Positive *V*_TD_ induces reverse-biasing of the graphene–QD junction. Increasing *V*_BD_ induces *n*-doping in the graphene channel and therefore increases the turn-on voltage of the diode. Inset: *IV* characteristic in logarithmic scale demonstrating diode leakage threshold around *V*_TD_=1 V. (**c**) *IV* characteristic of the graphene–CQD photodiode at different illumination intensities (values in the legend). Inset: *IV* characteristic in logarithmic scale demonstrating open-circuit voltage. (**d**) Responsivity and *EQE* of the graphene–CQD photodiode for illumination wavelength of 635 nm. Inset: band diagrams with and without *V*_TD_ bias. Black line is a guide to the eye.

To assess the origin of the enhancement of charge collection in the QD layer, we first operate the ITO–CQD—graphene as a photodiode, which enables us to independently measure the quantum efficiency of our device. To this end, we apply a *V*_TD_ bias across the PbS QD layer and measure the current *I*_TD_ as illustrated in the schematic of Fig. 3a, while floating the source V+ and V− contacts of the graphene. This structure operates as a photodiode in which a Schottky junction is formed at the graphene–QD interface. The diode characteristics are shown in Fig. 3b in dark and in Fig. 3c under optical illumination and confirm the formation of a Schottky junction at the graphene–QD interface, also evidenced by the presence of a built-in potential and the sustainment of open-circuit voltage under illumination. We measured the responsivity of the diode as a function of reverse applied bias. The quantum efficiency can be then directly calculated as:


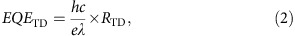


where *R*_TD_ is the photodiode's responsivity (Fig. 3d). In result, *EQE*_TD_ is measured to be ∼10% without any bias applied and increases with *V*_TD_ to about 75% at 1.2 V reverse bias, beyond which it saturates. The inset of Fig. 3d illustrates the corresponding band diagrams corresponding to the two regimes of operation as a function of the reverse bias *V*_TD_: for *V*_TD_=0 V the photodiode operates at short circuit conditions and charge collection is facilitated by a small part via carrier drift within the thin depletion region at the graphene–QD interface and by a large part via carrier diffusion within the large quasi-neutral region of the QD layer^24^. With progressively increasing *V*_TD_, the depletion region grows and thus enhances the efficiency of the charge collection. The *EQE*_TD_ value at saturation of 75% is estimated to approach the optical reflection limited value at the front PbS QD–ITO–air interface^23^,^25^,^26^, suggesting a nearly 100% charge collection efficiency.

### Phototransistor operation

**Figure 4:**
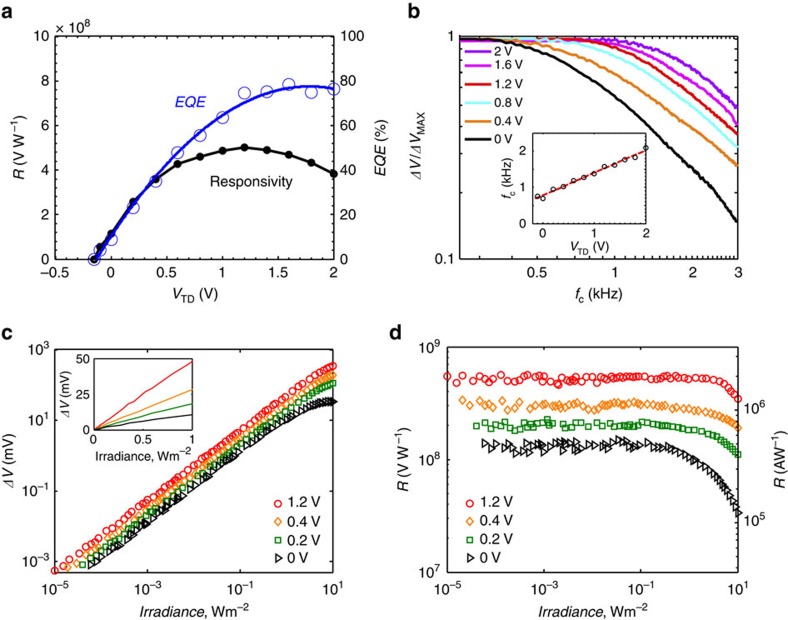
Visible/near-infrared phototransistor characteristics. (**a**) Responsivity and EQE of the visible/near-infrared phototransistor as function of applied *V*_TD_. Blue line is a guide to the eye. (**b**) Normalized photoresponse as a function of light modulation frequency for *V*_TD_ values specified in the legend. Light modulation frequency swept in the 250–3,000 Hz range. Inset: extracted 3 dB bandwidth values as a function of applied *V*_TD_ and used for *EQE* calculations. (**c**) Photo-induced signal as a function of incident irradiance. Lowest detectable irradiance of 10^−5^ W m^−2^ was measured at *V*_TD_=1.2 V. The linear dynamic range of the detector increases with increasing *V*_TD_ reaching over 110 dB at the optimal *V*_TD_. Selected *V*_TD_ values are shown in the legend. Inset demonstrates the linearity of photoresponse for high irradiance values. (**d**) Measured responsivity of the detector in V W^−1^ (left axis) and responsivity converted in A W^−1^ (right axis, see Methods) as a function of incident irradiance. Selected *V*_TD_ values are shown in the legend. The Illumination wavelength is 635 nm.

To quantify the impact of the *EQE*_TD_ enhancement on the performance and speed of the phototransistor operation, we measured the responsivity and time response of the phototransistor as a function of *V*_TD_, plotted in Fig. 4a,b. For the bias current *I*_SD_ of 100 μA, the responsivity reaches a value of 5 × 10^8^ V W^−1^ at *V*_TD_ of 1.2 V following the dependence of *EQE*_TD_ of the photodiode on *V*_TD_ (Fig. 3d). For a photodetector with a current read-out circuit this corresponds to a responsivity of 2 × 10^6^ A W^−1^ (see Methods). To corroborate our findings we also extracted the *EQE* of the phototransistor using the formula^27^ for responsivity (in units of VW^−1^) of photoconductive detectors:


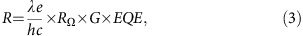


where *λ* the wavelength, *e* the elementary charge, *h* is the Planck constant, *c* the speed of light, *R*_Ω_ is the detector's resistance in dark and *G* is the photoconductive gain given by the ratio of carrier lifetime over the transit time. Here the carrier lifetime is governed by the time constant of the graphene–CQD–ITO photodiode response, in contrast with the case of the passive QD layer, in which carrier lifetime is determined by the sensitizing traps of the PbS QDs. To estimate the carrier lifetime *τ*_lifetime_ in our devices, we measured the photoresponse bandwidth of the phototransistor by sweeping the light modulation frequency *f*_c_ as demonstrated in Fig. 4b. The temporal response of the detector accelerates with increasing *V*_TD_, reaching an electrical 3 dB bandwidth of 1.5 kHz for the optimum *V*_TD_, which corresponds to an effective *τ*_lifetime_ of 106 μs. The time response of the graphene–CQD–ITO photodiode when measured independently yields a temporal response composed of two time constant components (see Supplementary Fig. 2): one of ∼1 kHz and a faster one on the order of 10 kHz. The overall time constant of the hybrid phototransistor results from the contributions of the two time decay components of the CQD photodiode, and both its bandwidth and gain are mainly determined by the slower component. The transit time *τ*_transit_ of the carriers traversing the graphene channel was calculated to be on the order of 1 ns by measuring the carrier mobility of the graphene (see Methods). This yields a photoconductive gain *G* on the order of 10^5^ and gain-bandwidth product of >1.5 × 10^8^ Hz. The extracted *EQE* of the phototransistor (shown in Fig. 4a) is in very good agreement with the directly measured *EQE*_TD_ of the photodiode. The responsivity follows the increase of *EQE* at lower values of *V*_TD_ and reduces at higher values of *V*_TD_. This is due to the accelerated response of the photodetector, resulting in a corresponding reduction of the gain. The hybrid photodetector achieves a concomitant demonstration of responsivity of 5 × 10^8^ V W^−1^, *EQE* of 75% and bandwidth of 1.5 kHz.

An equally important feature of a photodetector is its dynamic range for high-contrast applications such as remote sensing and imaging. We therefore showcase here the significant impact the transformation from a passive to an active sensitizing layer has on the dynamic range of the photodetector. The power dependence of the detector's photoresponse expressed in Δ*V* is illustrated in Fig. 4c. In these measurements, we change *V*_BD_ along with changing *V*_TD_ to keep the value of *R*_Ω_ at 3.7 kΩ, being the point of optimal photoresponse and highest DC transport mobility. Thus we keep the optimized values of (∂*g*/∂*n)* and *R*_Ω_ and compensate for the slight gating effect, induced by *V*_TD_ bias. Considering first the passively sensitized photodetector (at *V*_TD_=0 V the linear dynamic range is limited to 75 dB with an onset of photoresponse saturation at a power density of about 0.5 W m^−2^. With the application of *V*_TD_, we note a significant enhancement of the linear dynamic range of the detector to 110 dB at *V*_TD_ of 1.2 V. The high linear dynamic range is even more evident in the flat responsivity response across the wide range of power density, as shown in Fig. 4d. This is the result of having a photodiode as the sensitizing element instead of a passive sensitizer. In the latter case, the detector operates as a photoconductor whose dynamic range depends on the density of the sensitizing centres^14^,^21^,^28^. To assess the ultimate sensitivity of our detector, we performed independent noise measurements that yield D* of 1 × 10^13^ Jones at a wavelength of 635 nm, and 1 kHz light modulation (see Supplementary Fig. 3). The accompanied enhancement of the detector responsivity as a function of illumination wavelength is illustrated in Supplementary Fig. 4, following the typical absorption spectrum of PbS QDs with a bandgap of 1.2 eV.

**Figure 5:**
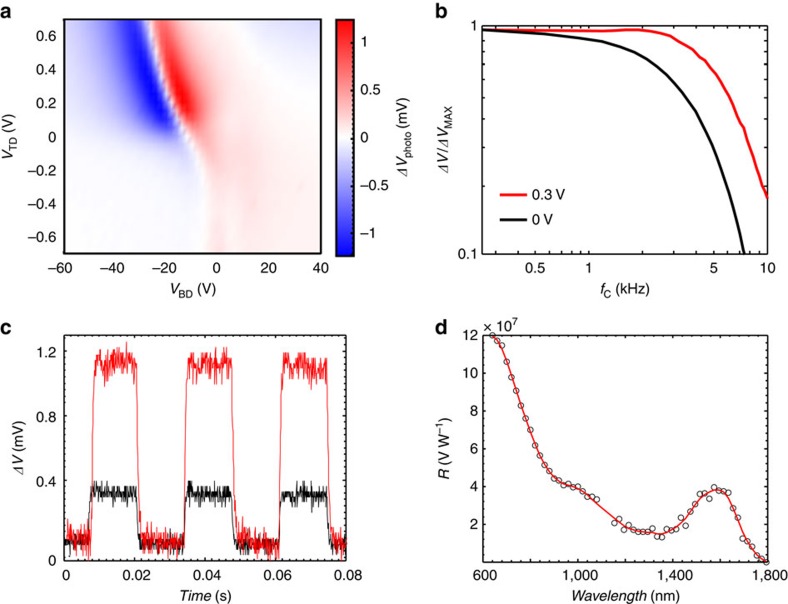
Visible/SWIR phototransistor characteristics. (**a**) Colour map of photovoltage response for a hybrid photodetector with large PbS CQDs as a function of the *V*_BD_ and *V*_TD_. Positive (red) and negative (blue) area correspond to injection of photo-generated holes into the *n*- and *p*-doped graphene. The optimal *V*_TD_ of 0.3 V results in a threefold increase of the photoresponse amplitude. Illumination is with 635-nm light and irradiance of 0.1 W m^−2^. (**b**) Photoresponse of the device as a function of the optical modulation. (**c**) Temporal response traces of the device at *V*_TD_=0 V (black) and *V*_TD_=0.3 V (red), demonstrating stable sub-millisecond photoresponse. (**d**) Spectral responsivity of the device with large PbS CQDs with exciton peak at 1,600 nm. The spectral sensitivity is determined by the absorption spectra of the quantum dots that can be readily tuned at their synthesis. The red line is a guide to the eye.

To showcase the power of our approach in extending the spectral coverage further into the short-wave infrared, where many applications require high sensitivity low-cost CMOS-compatible photodetectors, we report a phototransistor employing larger PbS QDs with absorption onset at 1.8 μm. The colour map in Fig. 5a shows the photoresponse of such device as a function of *V*_TD_ and *V*_BG_, revealing up to factor 3 enhancement when activating the charge transfer by *V*_TD_. Maximum photoresponse in this device was found at *V*_TD_=0.3 V. Detailed characterization of this device is described in Supplementary Note 1. The temporal response of the SWIR-sensitive photodetector is faster compared with the visible–near-infrared devices described above, with a measured 3 dB bandwidth exceeding 4 kHz (Fig. 5b,c). Figure 5d illustrates the spectral photoresponse of the detector at a reverse *V*_TD_ bias of 0.3 V, which shows that the responsivity of this device exceeds 1 × 10^8^ V W^−1^ in the visible range and 4 × 10^7^ V W^−1^ in the SWIR range. The calculated *EQE* is 14% for illumination wavelength of 1,600 nm and 35% for 635 nm, in both cases for the optimum *V*_TD_ of 0.3 V. This is a sixfold improvement compared with *V*_TD_=0 V.

## Discussion

We report a photodetector platform that integrates an electrically active colloidal QD photodiode architecture with a graphene transistor. The former allows the achievement of high charge collection efficiency and therefore *EQE*, as well as fast photoresponse, whereas the latter enables the simultaneous demonstration of ultra-high gain. The use of a photodiode instead of a passive sensitizing layer gives access to a much broader performance parameter space, as the time response and the gain of the phototransistor is now dependent to the temporal response of the photodiode and the carrier mobility of the graphene channel. It also allows the exploitation of additional carrier multiplication effects previously observed in QDs photodetectors and solar cells^29^,^30^ giving rise to the possibility of higher responsivity without sacrifice of bandwidth. This hybrid architecture finally illustrates the potential of graphene and other two-dimensional materials to be successfully integrated with other optoelectronic materials and open new ways for hybrid two-dimensional/zero-dimensional optoelectronics.

## Methods

### Graphene FET fabrication

Metal contacts were designed on a precleaned Si/SiO_2_ (285 nm) wafer using a direct laser writer lithography. Ti/Pd electrodes (2 nm/40 nm thickness) were e-beam evaporated at a base pressure 10^−3^ mbar and lifted-off in warm acetone bath.

CVD graphene grown on copper foil was transferred by a poly(methyl methacrylate) (PMMA)-assisted wet process onto the substrate with metal contacts. Ammonium persulfate was used to etch the copper substrate. After the transfer, the substrate with metal contacts and PMMA/graphene film was dried overnight at room temperature. PMMA was then removed by warm acetone, followed by annealing in forming gas at 300 °C to remove residues.

Laser writer lithography and Ar/O_2_ plasma etching were used to pattern the graphene layer. Graphene stripes had dimensions of 70 × 10 nm with 10 × 10 μm area between V+ and V− electrodes.

### PbS QD layer fabrication

The synthesis of PbS nanocrystals was carried out under inert conditions using a Schlenk line as previously described in literature^31^,^32^. The final PbS oleate-capped nanocrystals were dispersed in octane for device fabrication.

Electron beam lithography (EBL) was used to pattern 30 × 30 μm windows in PMMA resist at the centre of the graphene Hall bars. Desired PbS CQDs were then spin-coated right on the PMMA. Lift-off in warm acetone performed directly after spin-coating removed PMMA together with the excess of CQDs. As a result, the CQDs remained only in the areas defined by the lithography. The areas were designed to ensure isolation of the graphene channel from the ITO electrode introduced next and to fully cover the central region of the graphene Hall-bar.

PbS QD layers were fabricated by layer-by-layer spin-coating. For each layer, PbS solution (45 mg ml^−1^ in octane) was spin-cast onto the substrate at 2,500 r.p.m. for 15 s. Deposition of each layer was followed by spin-casting of EDT solution (2 vol.% in isopropanol) and two rinse-spin steps with octane and isopropanol. All the spin-coating steps were performed under ambient condition and at room temperature. The resulting thicknesses of nine PbS–EDT layers were 300±20 nm, as determined by a profilometer (Alpha-Step IQ).

### ITO electrode deposition

About 150-nm-thick ITO layer was DC sputtered (2 mTorr, 60 W) to form a transparent electrode on top of the PbS layer. EBL lithography allowed to form the overlapping area of ITO, CQD and graphene precisely between the V+ and V− electrodes and avoid shortening of ITO and graphene channels. Each fabricated chip contained up to seven devices with top-electrodes.

### Electrical characterization

All transport measurements were carried out at room temperature, in a shielded enclosure and vacuum (10^−3^ mbar) to improve the device stability. Device illumination was done through a viewport by a 635 nm wavelength diode laser, modulated with a mechanical chopper or turned on and off. The laser was coupled to a digital variable optical attenuator to provide output power tunability. In addition, a supercontinuum Vis/NIR laser (SuperK Select) was used to measure photoresponse spectra. Ithaco 1211 amplifier was used to measure *I*_TD_. Ithaco 1201 and Stanford SR810 amplifier were used for voltage read-out of photoresponse in combination with a mechanical light chopper with 250–3,000 Hz range.

We utilized NI DAQ 6230 as a voltage and TTI PLH250 as a current source in our measurements. For photovoltage read-out, a constant current of 100 μA was driven between the source and the drain probes, while V+ and V− probes were used to measure the differential voltage drop in the active area of the device.

### Responsivity conversion

Directly measured values of responsivity in V W^−1^ can be converted into A W^−1^ for convenient comparison to photodetectors with current-based read-out and different bias using the formula:


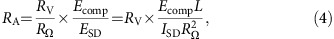


where *R*_V_ is the responsivity in V W^−1^, *E*_SD_ is the electric field, *L* is the length of active area, *I*_SD_ is the source–drain current and *R*_Ω_ is the resistance of our detector. *E*_comp_ is the electric field value used for comparison. Values presented in Fig. 4d were calculated for *R*_Ω_=3.7 kΩ, *I*_SD_=100 μA, *L*=10 μm and *E*_comp_=5 kV cm^−1^.

### Transconductance, mobility and *τ*_transit_ calculations

We calculate the transconductance of our phototransistor (∂*g*/∂*n*), where *n* is the charge carrier concentration in graphene, by taking a derivative of the *R*_Ω_ (*V*_BD_) curve. Capacitive coupling of the 285-nm-thick SiO_2_ gate is 7 × 10^10^ cm^−2^ V^−1^.

Charge carrier mobility *μ* is then calculated for the positive (electron) and negative (hole) transconductance maximums using: ∂*σ*/∂*n*=*e* × *μ*, where *e* is elementary charge. Maximum transconductance of 2.4 S cm^2^ corresponds to 1,500 cm^2^ V^−1^ s^−1^ of electron mobility in graphene.

Transit time *τ*_transit_ of charge carriers in the graphene channel is calculated as:


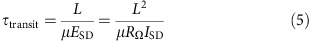


### Data availability

The data that support the findings of this study are available from the corresponding authors upon request.

## Additional information

**How to cite this article**: Nikitskiy, I. *et al.* Integrating an electrically active colloidal quantum dot photodiode with a graphene phototransistor. *Nat. Commun.* 7:11954 doi: 10.1038/ncomms11954 (2016).

## Supplementary Material

Supplementary Information Supplementary Figures 1-5 and Supplementary Note 1
