# Supplementary material for: Integrating an electrically active colloidal quantum dot photodiode with a graphene phototransistor
Source: Nat Commun. 2016 Jun 17;7:11954. doi: 10.1038/ncomms11954 (PMC4915030; doi:10.1038/ncomms11954)
Supplement: Supplementary Information — Supplementary Figures 1-5 and Supplementary Note 1 [file ncomms11954-s1.pdf]

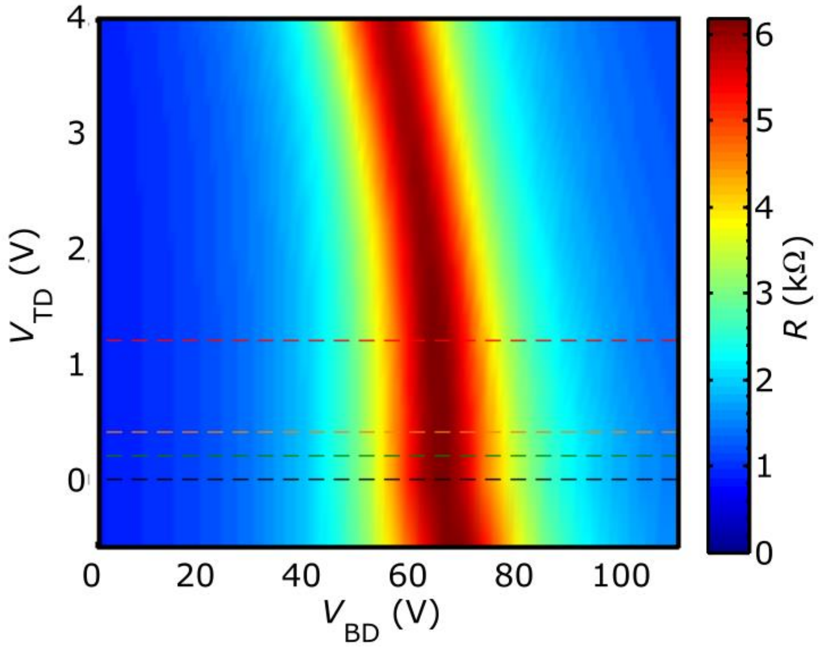

Supplementary Figure 1. Gate dependence of device's resistance. Color map of the resistance of the phototransistor's active area. Dashed lines serve as a guide to the eye and are located at V_{TD} values of 0 V (black), 0.2 V (green), 0.4 V (orange) and 1.2 V (red) correspondingly. V_{TD} range of 0 V - 1.5 V demonstrates only minor shift of the Dirac peak in V_{BD}. $V_{TD} < 0$ V and $V_{TD} > 1.5$ V induces prominent shift of the Dirac peak and corresponds to strong I_{TD} leakage appearing in CQD layer. High I_{TD} current suppresses the photoconductive gain and phototransistor demonstrates poor photoresponse this ranges.

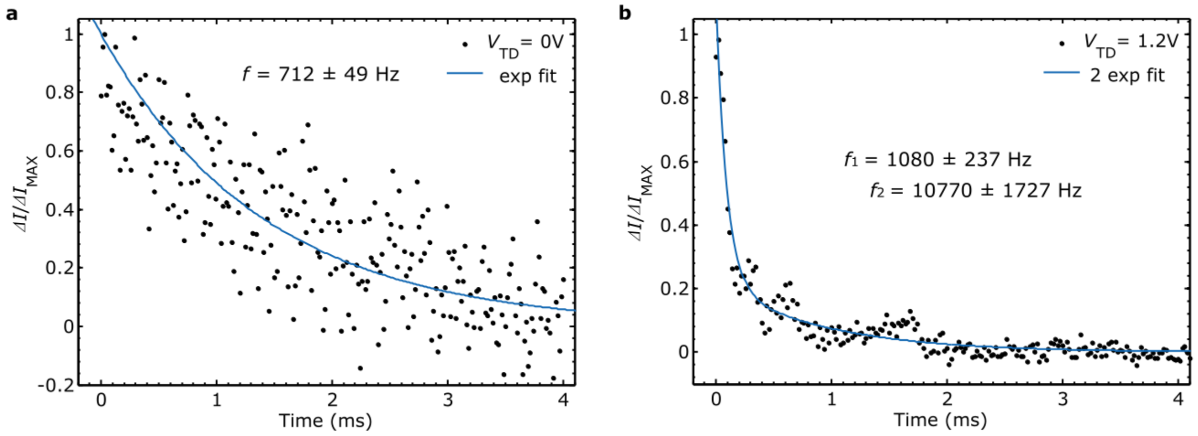

Supplementary Figure 2. Temporal response of Graphene-CQDs-ITO photodiode. Temporal response of the photodiode extracted by measuring the photo-induced current change between the ITO top-electrode and graphene drain electrode (**a**) without and (**b**) with applying voltage on the top electrode. Source electrode was kept floating at all time. Blue lines represent the fits of the photocurrent decays with the exponential functions. In the case of V_{TD} = 0 V (**a**) the decay was found to have single component with a cut-off frequency of 712 ± 49 Hz. At V_{TD} = 1.2 V (**b**) the temporal response is best fitted with a double exponent function and yields two components with cut-off frequencies of about 1 kHz and 10 kHz. Appearance of the faster component indicates that temporal response of the detector is controlled by the application of V_{TD} bias.

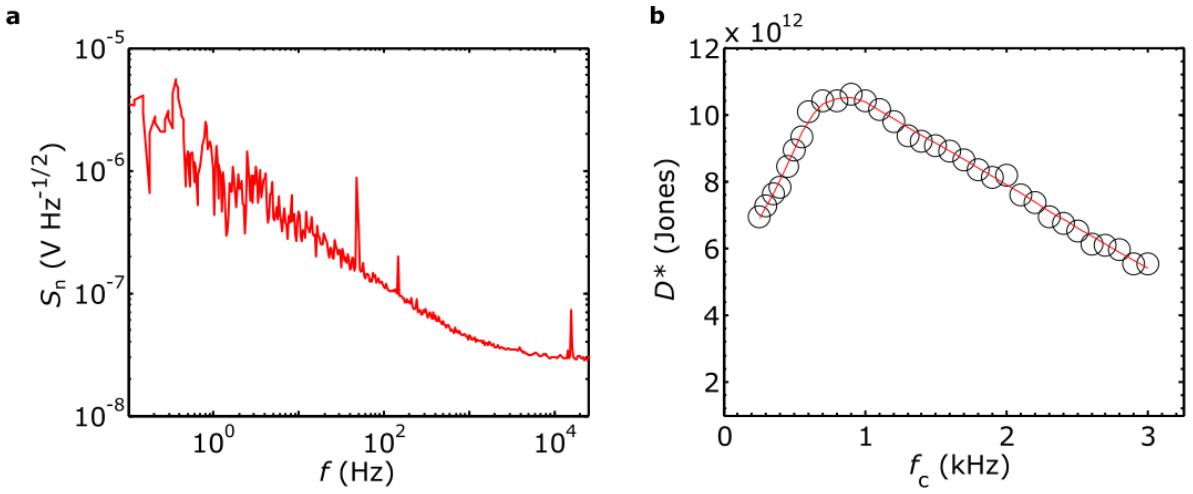

Supplementary Figure 3. Noise spectra and detectivity. a, Amplitude spectral density of noise of the phototransistor device. Spectra yields from a Fourier transform of a 100-second-long traces of voltage drop fluctuations in the device's active area measured in dark, at I_{SD} = 100 μA, V_{BD} =75 V, V_{TD} = 1.2 V. Amplifier bandwidth is 250 kHz. Noise spectra shows that in total device noise 1/f noise of graphene channel dominates at lower frequencies and white noise of the setup dominates at higher frequencies. **b**, Specific detectivity D* of the phototransistor as a function of light chopping frequency. For our best devices with responsivities of 5×10^8 V W^{-1} the D* values are in the range of 1×10^{12} -1 × 10^{13} Jones depending on the noise level. We utilize the noise spectra and frequency dependence of the photoresponse to extract D* dependence on the light chopping frequency. The optimized value exceeds 1×10^{13} Jones at $f_c \sim$ 1 kHz.

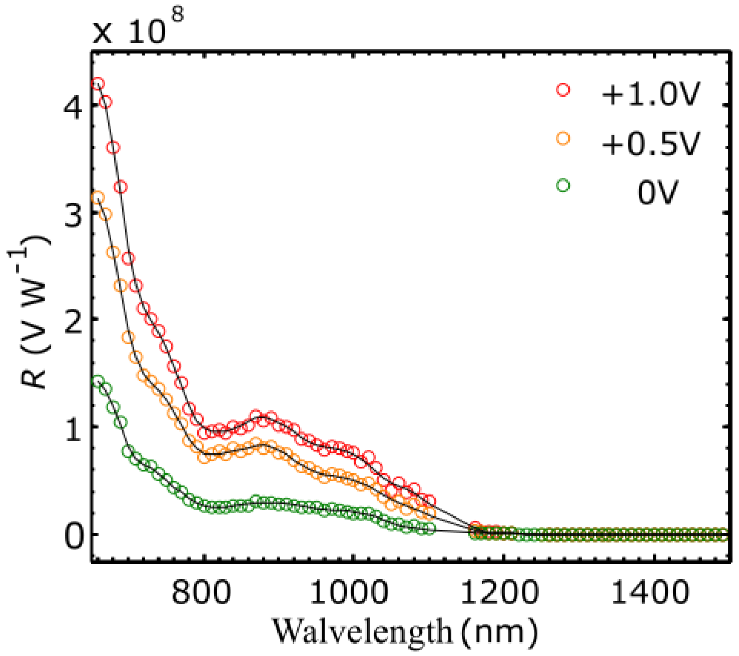

Supplementary Figure 4. **Photoresponse spectra.** Spectra of the Vis/NIR photodetector's response at different applied V_{TD} (values in the legend). Black lines are guides to the eye. Obtained spectral dependences of the photoresponse follow the absorption of the CQDs with the exciton peak around 900 nm. The shape of the spectra is similar at different V_{TD} values, which confirms that demonstrated enhancement of phototransistor's response by application of V_{TD} takes place for any detected wavelength of light. Black lines are guides to the eye.

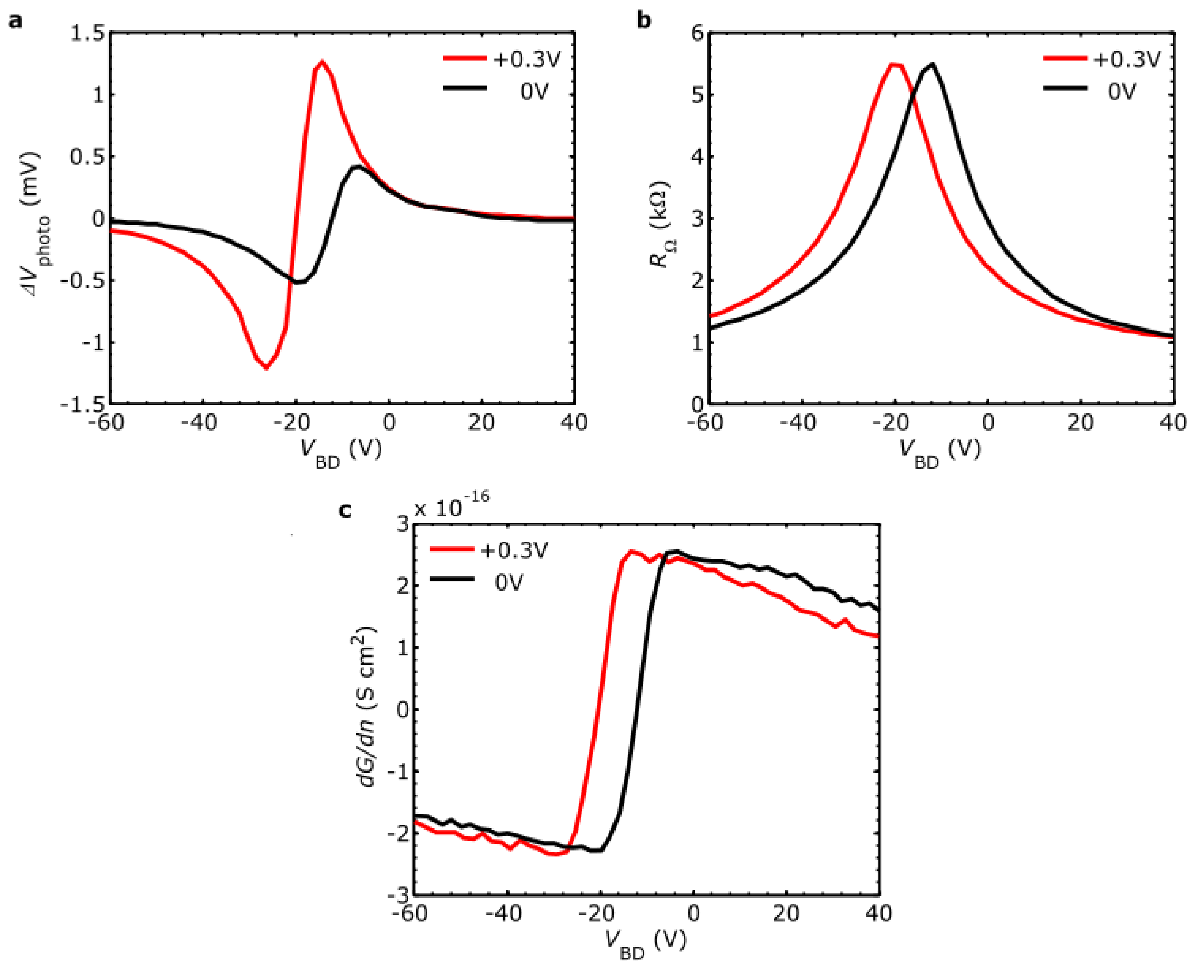

Supplementary Figure 5. **Characterisation of Vis/SWIR photodetector.** Backgate dependence of **(a)** photoresponse **(b)** resistance **(c)** transconductance of the Vis/SWIR phototransistor with and without applied V_{TD} (values in the legend).

Supplementary Note 1. Characterisation of the Vis/SWIR photodetector

The graphene channels of the Vis/SWIR photodetector devices were fabricated in the same shape as in the case of Vis/NIR detectors, but covered with a layer of larger CQD that can absorb light in Vis, NIR and SWIR range with wavelength up to 1800 nm. Active device area is 10 × 10 µm^2 and CQD layer thickness is 300 nm. The layer of this type of CQDs exhibited low leakage threshold. Response to light of the phototransistors was found to be maximum at V_{TD} = 0.3 V, about 3 times stronger as compared to operation with no applied V_{TD} (Supplementary Figure 5a). Further increase of V_{TD} bias, as well as application of negative V_{TD}, induces strong I_{TD} leakage current and suppresses the photoconductive gain. Unlike in the Vis/NIR phototransistors, in these devices V_{TD} induces strong gating of the graphene channel. This results in a significant shift of resistance and transconductance peaks due to the high dielectric permittivity of the CQD layer formed by smaller size PbS nanocrystals (Supplementary Figure 5b,c). However, we keep track of this shift when comparing the photoresponse at different V_{TD} values. The maximum transconductance of the detector stays unchanged ≈ 2.4 S cm^2, which corresponds to and electron mobility of 1500 cm^2 V^{-1} s^{-1} in graphene. Thus, photoresponse enhancement is due to increased charge collection efficiency under applied V_{TD}. Acceleration of temporal response results in electrical 3 dB bandwidth value change from 2.3 kHz to 4.6 kHz. This yields an *EQE* increase from 6 % to 35 % at 635 nm illumination wavelength and from 2.4 % to 14% at 1600 nm, as calculated for I_{SD} = 100 µA, R_Ω = 4.5 kΩ and μ=1500 cm^2 V^{-1} s^{-1}.
